# Effects of Varying Nano-Montmorillonoid Content on the Epoxy Dielectric Conductivity

**DOI:** 10.3390/molecules29194650

**Published:** 2024-09-30

**Authors:** Yujia Cheng, Guang Yu

**Affiliations:** Mechanical and Electrical Engineering Institute, Zhongshan Institute, University of Electronic Science and Technology of China, Zhongshan 528400, China; chengyujia@zsc.edu.cn

**Keywords:** nano-MMT, epoxy, dielectric conductivity

## Abstract

This study investigates the correlation between the interface structure and macroscopic dielectric properties of polymer-based nanocomposite materials. Utilizing bisphenol-A (BPA) epoxy resin (EP) as the polymer matrix and the commonly employed layered phyllosilicate montmorillonoid (MMT) as the nanometer-scale dispersive phase, nano-MMT/EP composites were synthesized using composite technology. The microstructure of the composite samples was characterized through XRD, FTIR, SEM, and TEM. Changes in the morphology of the nanocomposite interface were observed with varying MMT content, subsequently impacting dielectric polarization and loss. Experimental measurements of the dielectric spectrum of the nano-MMT/EP were conducted, and the influence of the material interface, at different nano-MMT contents, on the dielectric relaxation was analyzed. The study delves into the effect of the nanocomposite interface structure on ion dissociation and migration barriers, exploring the ionic conductivity of nano-MMT/EP. Lastly, an analysis of the impact of different nano-MMT contents on the dielectric conductivity is presented. From the experimental results, the arranging regularity of polymer molecules in the interface area raises. In the matrix, the ion migration barriers decrease significantly. The higher the MMT content in the interface, the lower the migration barrier is. Until the MMT content exceeds the threshold, the agglomerated micro-particles form, which decreases the polymers’ space distribution regularity, and the ions migration barrier raises. According to the changes in the rule of the ions migration barrier with the composite interface structure content, the reason for dielectric conductivity changes can be judged.

## 1. Introduction

Polymer-based nanocomposite dielectric materials consist of a blend of polymer matrix and nanoparticles [1,2,3]. Generally, the addition of a small amount of the nanoscale dispersive phase can significantly enhance the properties of the composites. In the realm of nanocomposite dielectric systems [4,5], several noteworthy research directions emerge, seeking answers to the following questions:

1. How does an interface form based on the interaction between the two phases?
2. What role do the surface characteristics of the dispersed phase play in the process of interface formation?
3. What is the impact of internal charges at the interface on the macro properties of the materials?
4. How can the regulation of charge distribution enable nanodielectric applications in diverse areas?

**Figure 1:**
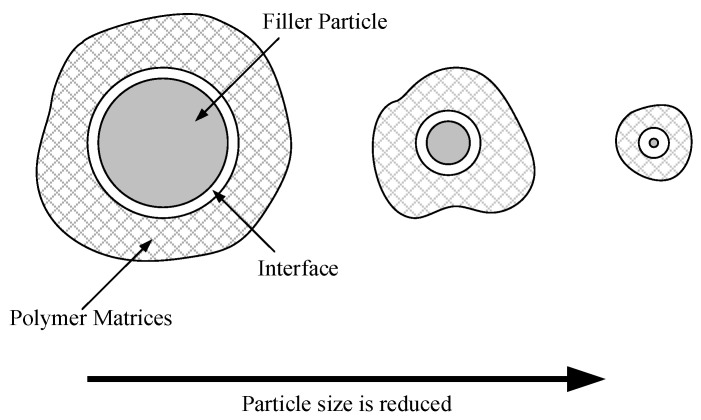
The schematic illustrates an increase in interface volume as the size of the dispersion phase decreases.

Exploring the interface structure and behavior through research reveals the reasons behind property changes [6,7]. For instance, the surface characteristics of nanoparticles can alter the local structure and charge distribution of polymers. As the spatial dimension of the dispersed phase decreases, the relative volume of the interfacial structure gradually increases [8,9,10]. In this context, increased molecular motion at the interface gradually becomes the dominant factor influencing performance, as illustrated in Figure 1.

The nanoparticles’ surface at the interface can trap free charges, restricting the shift of bound charges within a smaller space. This results in a decrease in both the mobility of the free carriers and the energy from the electric field to a certain degree [11,12]. The likelihood of matrix destruction owing to carrier impact is reduced, and the accumulation of charges in the electrodes is strengthened, thereby diminishing the electric field intensity at the interface. Higher field strengths are required to inject charges into the electrodes, consequently increasing the macro short-term breakdown voltage of the dielectric [13,14]. The nanocomposite’s properties are special. It is because of the unique nanocomposite interface structure. When the system scale is shrunk to the typical phase coherence scale, the nanocomposite interface forms. Because of the quantum coherence, the observed physical property appears to be a significant quantum mechanical effect. In principle, according to the electronic De Broglie wavelength, mean free path, relaxation time, and phase coherence length of nanoparticles, the particles’ quantum behavior can be analyzed qualitatively.

With the ongoing advancement of characterization techniques and testing instruments, particularly the widespread application of computer simulation technology in scientific research, innovations have emerged in the study of nanodielectric properties [15,16,17]. Conduction in a solid dielectric is classified into ionic and electronic conduction based on carrier species. Ionic carriers, having a relatively larger volume, are less influenced by localized energy states in their migration path, making ionic conduction easier compared to electronic conduction. However, in an inhomogeneous medium, structural deficiencies can capture migrating ionic carriers, leading to ion accumulation in localized states and subsequent space-charge accumulation. This distortion in the electric field results in partial discharge. According to XRD, FTIR, SEM, and TEM, the composite samples deal with microstructure characterization. From the test result, some kinds of nanoparticles show good compatibility with the EP matrix. These nanoparticles disperse uniformly in a resin matrix, which will form nanocomposites [18].

In polymers, electronic carriers are primarily generated by stimulated emissions at the localized state level rather than intrinsic excitation of the medium molecules [19]. The polymer matrix exhibits aggregation-state structural characteristics, with coexisting crystalline and indefinite states. Electronic hopping conductivity occurs as carriers move repeatedly between traps and out of the traps. Therefore, the carrier concentration is determined by the shallow trap density in nanocomposites, while the electron mobility is influenced by the deep trap density. The breakdown field strength of a dielectric is a crucial factor in the application of structural insulation materials, and extensive research has been conducted on the breakdown properties of nanocomposites. The short-term breakdown field strength increases with higher nanophase content, according to Tanaka’s multicore model [20]. However, different experiments show varying trends due to the formation of defects at the matrix interface when nanoparticles are added. These defects possess energy levels that capture migrating electrons, shortening the carrier-free path. Under an external electric field, the reduced energy accumulation lowers the probability of electron impact ionization. Long-term breakdown studies of nanocomposites have focused on the impact of space charge behavior on partial discharge, electrical tree, and corona resistance properties [21]. Some researchers have analyzed the long-term breakdown properties of the materials with a nanocomposite interface model, attributing changes in macro-properties to electron transport behavior in the interface microdomain [22]. Discrepancies in experimental results highlight the unclear influence of the nanocomposite interface on properties. Particularly, further research and discussion are needed to understand the effects of adding nanoparticles with different contents on the dielectric properties of composites.

## 2. Preparation of Nano-MMT/EP

Whether in fundamental research or industrial application, polymer/layered silicates (PLSs) are used widely. Among them, MMT is the most representative phyllosilicate. PLS nanocomposites are different from other nanocomposites regarding the preparation methods, because the PLS nanocomposites are prepared using unique intercalation composite technology. The intercalation composite means the monomer or polymer molecules are inserted into the nano-space between different silicate clay. The nano-MMT/EP is scattered over the polymer matrix in the form of intercalation or peeling. Therefore, in this study, the nano-MMT particles were intercalated into EP. According to the relative references, the MMT contents of 1%, 3%, 5%, and 7% are selected for comparative analysis.

The used reagents in these experiments are shown in Table 1.

The used equipment in these experiment are shown in Table 2.

### 2.1. Organic Modification of MMT

Untreated montmorillonoid (MMT) exhibits hydrophilic properties and displays weak compatibility with polymers, in contrast to the uniform particle dispersion observed in the matrix resin. Therefore, the organic modification of MMT plays a crucial role in the preparation of nanocomposites. The essential principles of organic modification involve the exchange of cations within the interlayer of MMT using organic quaternary ammonium salt. This results in an ion-exchange reaction, covering the MMT surface with organic groups and transforming its surface properties from hydrophilic to lipophilic (Equation (1)).

CH_3_(CH_2_)_n_NR_3_X + M–MMT → CH_3_(CH_2_)_n_NR_3_–MMT + MX (1)

(R: H, CH_3_; X: Cl, Br, I; M: Na^+^, Ca^2+^, Mg^2+^).

In this study, we utilized natural Na-MMT with a cation exchange capacity of 80 mmol/100 g. Following purification, the MMT was mixed with deionized water and was then placed in a three-necked bottle, where it was stirred to produce a turbid liquid. The corresponding OTAC quality was determined based on the MMT ion-reaction exchange capacity. A solution was prepared by adding deionized water and was then added dropwise to the turbid liquid.

Due to the organic modifier being a long-chain organic ammonium salt, its molecular chain activity is insufficient at low temperatures, preventing it from entering the MMT layers through molecular motion. Therefore, the mixture necessitated stirring at 80 °C to ensure a complete cation exchange reaction. The resulting mixture underwent repeated washing and filtration with deionized water. Suction filtration was carried out until 1% silver nitrate titration indicated no presence of a white precipitate. After drying, grinding, and filtration through a 400 mesh, organic MMT was obtained.

### 2.2. Nano-MMT/EP Sample Preparation

**Figure 2:**
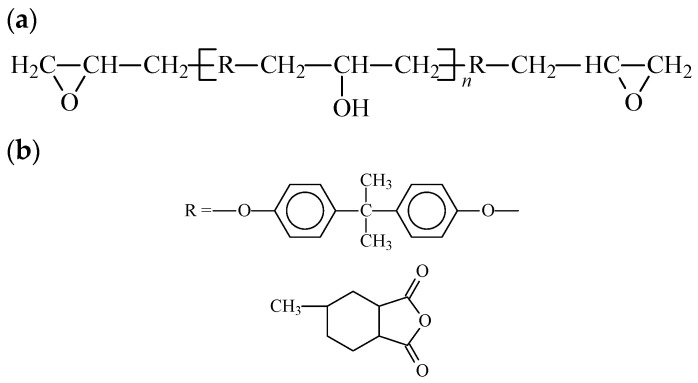
Illustration of simplified forms of (**a**) the E-44 monomer and (**b**) 3-MHHPA.

There is a wide range of EP (epoxy) and curing agents available. Various EP monomers were paired with curing agents to create cured EP resins with distinct properties. In this study, the widely utilized bisphenol-A EP (E-44) served as the monomer, and 3-MHHPA was chosen as the curing agent (Figure 2). These components were blended with organic MMT, resulting in the preparation of nano-MMT/EP samples.

EP monomers were measured in moderate quantities, and the viscosity decreased following preheating. Subsequently, the required mass ratio of organic MMT was determined after drying. The EP and MMT particles were directly mixed at a temperature of 80 °C, facilitating the blending process at low viscosity and aiding in the dispersion of MMT particles in the resin. After thorough stirring, the MMT particles were integrated into the resin, albeit with a notable introduction of gas due to mechanical stirring.

The temperature was maintained, and the solution was degassed until the melt achieved transparency. Subsequently, at 80 °C, the degassed melt was combined with acid anhydride, and the curing reaction promoter was introduced. The mixture underwent stirring until homogeneity. After decompression and air extraction, any remaining air bubbles were eliminated, as their persistence could result in air gaps or holes in the cured samples. Consequently, the air extraction process was reiterated until the system was completely free of air.

Following the aforementioned treatment steps, the blended melts were poured into a mold coated with vacuum silicone grease. Through procuring and curing at 80 °C, the mixture underwent consolidation. Finally, the nanocomposite samples were prepared through natural cooling and pattern drawing.

## 3. Structure and Morphology Characterization of Nano-MMT/EP

### 3.1. FTIR Analysis of MMT before and after Organic Treatment

The MMT layers contain exchangeable cations that serve to balance the lamellar electrostatic potential [23]. Following organic treatment, the free cations are replaced by organic ammonium ions, re-establishing electrostatic equilibrium between the MMT lamella and organic cations. This process creates a steady electrostatic force. The organic cations and negative charge centers in the lamella undergo simple harmonic vibration in their respective equilibrium positions. When the energy of specific-wavelength infrared rays aligns with the vibrational level, it triggers vibration, resulting in FTIR signal generation.

**Figure 3:**
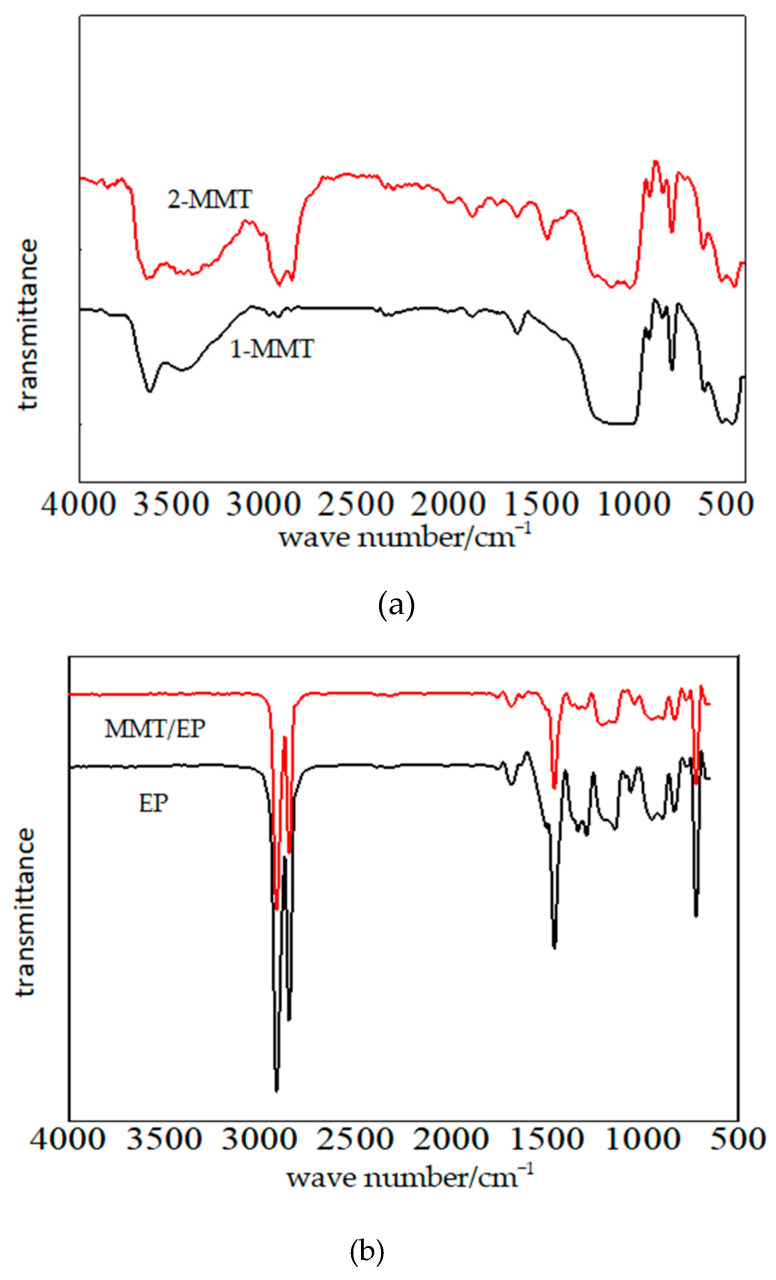
FTIR patterns for MMT, MMT/EP, and EP samples. (**a**) FTIR patterns for MMT samples, (**b**) FTIR patterns for MMT/EP and EP samples.

FTIR spectroscopy was employed to analyze MMT particles before and after organic treatment, confirming the formation of electrostatic equilibrium between the organic modifier and lamella. Additionally, it verifies whether the long-chain molecules of the modifier can enter the lamellar spaces and undergo pre-intercalation. The Bruker CZ304 FTIR spectrometer continuously scanned MMT samples in the 500–4000 cm^−1^ wavenumber range before and after organic treatment. By observing the changes in the infrared spectrum absorption peaks, one can analyze how the MMT lamellar structure is altered due to the organic treatments. In nano-MMT/ER, the nano-MMT particles experience surface modification by octadecyl quaternary ammonium salt. The MMT particles before and after modification are represented by 1-MMT and 2-MMT. The FTIR test results of 1-MMT and 2-MMT are shown in Figure 3. From Figure 3a, the characteristic peaks position of 1-MMT and 2-MMT are basically identical. Around 1100–600 cm^−1^, the bending vibration peaks of the Si-O bond and Al-O bond exist. Around 1640 cm^−1^, the Na^+^ characteristic peak exists. According to a literature review, the lamellar structure of MMT before and after modification does not change. From the FTIR curve of 2-MMT in Figure 3a, the sharper -CH3- and -CH2- asymmetrical stretching vibration absorption peaks exist around 2855~2978 cm^−1^, so the cation exchange reaction happens between Octadecyl ammonium chloride with MMT particles, from which the stable bonding interaction forms.

From Figure 3b, there are three characteristic absorption peaks with high energy in the FTIR pattern of pure ER. They are located at 723 cm^−1^, 1450 cm^−1^, and 2850 cm^−1^. They correspond to the rocking vibration peak, bending vibration peak, and symmetrical and unsymmetrical stretching vibration peak of the C-H bond, respectively, two split peaks exist at around 2850 cm^−1^. Furthermore, a bending vibration peak of the C-H bond exists at around 719 cm^−1^. Around 860 cm^−1^, the methylene symmetrical and unsymmetrical low energy bending vibration peak exist.

According to the FTIR test results in Figure 3b, after the nano-MMT particles are added, the vibration absorption peak value of the composite decreases; thus, the hydrogen bond forms between the nanoparticles with the polymer matrix, which affects the intermolecular binding force. The original bond force constant changes, from which the values of the stretching vibration peak and bending vibration peak both decrease. The strength of the absorption peak decreases.

### 3.2. Nanophase Dispersion Morphology Observation via EM

To ensure effective nanocomposite interface formation in nano-MMT/EP, it is crucial to achieve uniform dispersion at the nanoscale. The dispersion state in the matrix was directly observed through electron microscopy (EM), a widely employed technique for detecting micro-morphology in PLS nanocomposites. In this study, transmission electron microscopy (TEM) and scanning electron microscopy (SEM) were employed to observe MMT dispersion and composite fracture surfaces. The sample surface underwent carbon coating sputtering before analysis.

**Figure 4:**
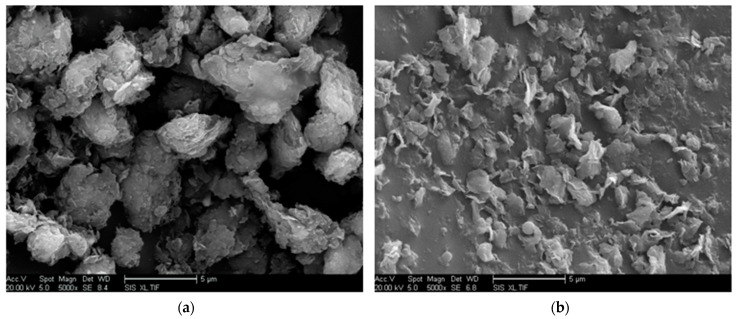
TEM image depicting various forms of MMT dispersion at the same magnification. (**a**) Unexfoliated MMT, (**b**) exfoliated MMT.

**Figure 5:**
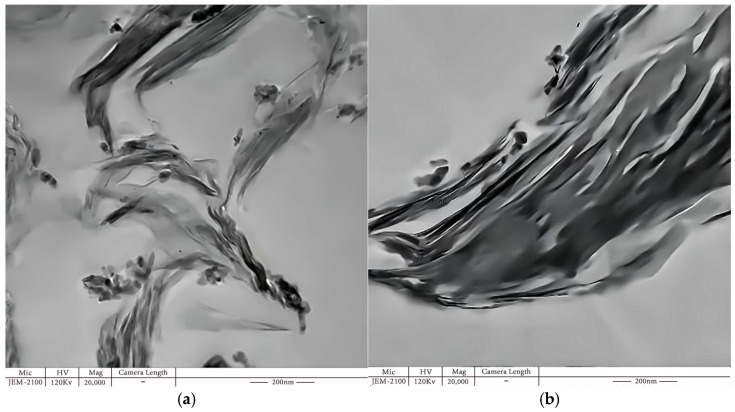
SEM image showing diverse MMT dispersion statuses at the same magnification. (**a**) Agglomeration, (**b**) homodispersion.

Both intercalated and exfoliated MMT were observed, and the uniform dispersion of nano-MMT particles in the matrix was determined (Figure 4 and Figure 5). In Figure 4a, the MMT nanoparticles within the resin matrix maintained their inherent configuration, dispersed as particles. Figure 4b reveals an evident exfoliated structure of the MMT nanoparticles.

SEM allows for an intuitive observation of the nano-MMT particle dispersion morphology. A uniform dispersion is noted in Figure 5, evident from the SEM image where the boundary between the nano-MMT particles and the matrix resin appear blurred, and the coverage distribution is homogeneous. Conversely, if MMT cannot be peeled and particle agglomeration occurs, the interfacial interaction weakens, resulting in a clear boundary and an inhomogeneous coverage. Local particle accumulation was observed.

## 4. Composite Relaxation Polarization and Dielectric Losses

According to the intensity of materials polarity, EP belongs to a polar polymer. Under the applied electric field, the polarization in the EP curing system is mainly electronic polarization and dipoles orientation polarization. Among them, the dipoles orientation polarization is dominant. In regard to montmorillonite crystals, the polarization is mainly electronic polarization, ionic elastic displacement polarization, and thermion polarization, because the electron cloud in the MMT layers will be restricted by atoms in unit cells nodes. The collaborative binding effect forms, so the ionic elastic displacement polarization and thermion polarization are dominant.

**Figure 6:**
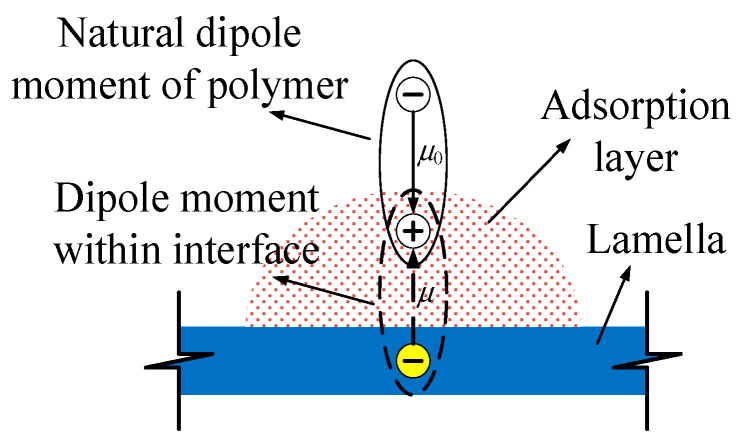
Schematic graphs for interface restriction to polarization.

When the Interface layers form between EP and MMT composite, the polarization in the composite system will change. The dipoles orientation polarization of EP and ionic elastic displacement polarization and thermion polarization of MMT layers are restricted by electrostatic attraction in the composite interface, so they are difficult to establish. This principle is shown in Figure 6. In EP noumenon, the dipoles orientation polarization is restricted by a crosslinking structure and molecular segmental mobility, but it can be established to a certain extent. Similarly, in MMT noumenon, the crystal lattice will cause thermion polarization under the effect of thermal motion, which presents the relaxation polarization characteristics, but in the composite system, when there is an EP and MMT composite, the interface adsorptive layer appears, as shown in Figure 6. Relying on electrostatic force, the unlike charges center in phase two and adsorb mutually in the interface, so the intrinsic polarization in phase two will be restricted. On the one hand, the EP doublet steering in the applied electric field is restricted. On the other hand, the thermionic polarization of the MMT crystal lattice is restricted. Furthermore, in the movement of the dipole moment both ends are also restricted by molecules in the two phase system. Therefore, the polarization is difficult to establish.

The nano-MMT composite interface is introduced into EP. The intensity of the polarization in the composite strengthens, which mainly benefits from the restriction of the composite interface on the polarization effect in the two phase system, but this restriction on the relaxation polarization process can only be strengthened in the nanocomposite system. If the two phases cannot be composite in the nanoscale, this illustrates that EP cannot enter the MMT layers of the composite, so the composite interface will greatly reduce.

With the condensation polymerization process of EP generating a curing system, the nanocomposite interface forms gradually. Different cure temperatures cause the polymer molecule to present different movement characteristics, from which the interface layer size and interfacial adsorption strength will also change. Finally, each polarization establishment process in the composite system are affected. The polycondensation of EP and acid anhydride type curing agents generates a crosslinking structure, of which the process is divided into a precuring reaction and a curing reaction. Firstly, the precuring reaction arises at a low temperature. At the beginning of the curing reaction, the acid anhydride initiates the EP polymerization, which generates the polymerization centers. Microgel forms in the EP monomer molecule. With the reaction process proceeding, the sol part in the microgel system gradually decreases. The gel part increases gradually until the gel polymer forms. The different gels interact via Van Der Waals force. In this process, the EP monomer molecules are mainly composite with the MMT layers. A large amount of heat will release in the EP condensation polymerization process. This heat of the polymerization reaction diffuses from the polymerization center gradually. Furthermore, it is absorbed by the monomer molecule and the MMT layer, from which the molecular thermal motion is excited. In this process, the monomer molecules show good compatibility with alkyl ammonium salt. They can enter into MMT layers and be adsorbed, but this adsorption is unstable. On the one side, the heat of the polymerization reaction makes the athletic ability of the monomer molecule stronger, which makes it difficult to achieve stability with the MMT layer. On the other side, comparing with the crosslinking structure with cured epoxy resin, the size of the monomer molecule is small. The probability of these molecules entering into the layers is high. The lamella will adsorb more monomer molecules, which causes average effect intensity between the monomer molecules with a decrease of the lamella. The electrostatic force in the interface layer decreases. In such a weak interaction, the interface structure can be destroyed easily by molecular thermal movement. In the precuring process, plenty of EP monomer molecules are consumed, from which the microgels form. The curing reaction is carried out between these microgels at a higher temperature, from which the crosslinking structure forms. The composite interface structure mainly forms in this process. When the monomer molecules gradually become gels, the thermal movement level of these gels reduces. Furthermore, the size of the gels is larger than that of the monomer molecules, the quantity of gel being adsorbed by the MMT layer is less, the composite interface strength is higher, and the adsorption is closer.

**Figure 7:**
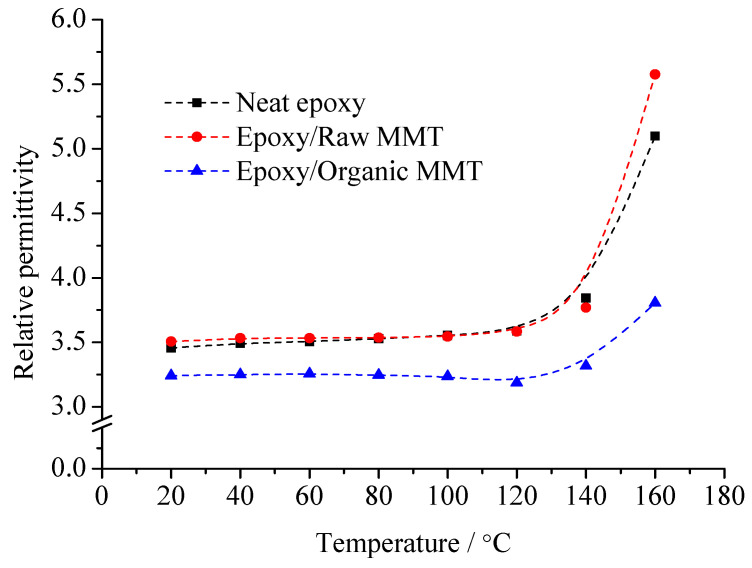
Temperature dependence of relative permittivity of various compound specimens.

**Figure 8:**
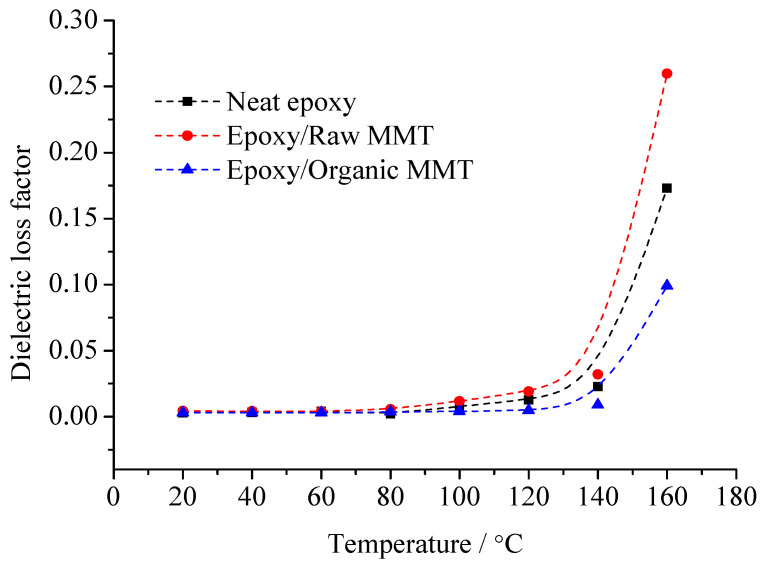
Temperature dependence of dielectric loss factor of various compound specimens.

The pure EP cured epoxy resin, raw MMT/EP, and organic MMT/EP samples are prepared. The sample relative dielectric constant, *ε*_r_, and dielectric loss factor, tan*δ*, at different temperatures were measured, from which the information of the dielectric polarization establishment process was obtained. The effect of the interface on the polarization process was considered. With the temperature changes, the *ε*_r_ and tan*δ* of the three samples are shown in Figure 7 and Figure 8.

From Figure 7, no matter whether at low temperature or high temperature, the dielectric constant of organic MMT/EP is the lowest. With the temperature increasing, the dielectric constant growth rate of organic MMT/EP is slower than that of EP and raw MMT/EP. Among them, the dielectric constant of raw MMT/EP is slightly higher than that of pure EP. Especially if the temperature exceeds 140 °C, they are significantly different.

The change rule of the three samples of dielectric loss factor with temperature is shown in Figure 8. From this figure, with the temperature rising, all of the samples’ losses increase. In the range of 20~60 °C, the three samples’ losses demonstrate little difference. When the temperature exceeds 60 °C, the differences increase gradually. No matter whether at low temperature or high temperature, the tan*δ* value of organic MMT/EP is the least. The tan*δ* value of raw MMT/EP is the maximum.

According to the difference of the three samples’ mesostructure, the reason for the dielectric constant and dielectric loss changes can be derived. At low frequency, the relaxation polarization of pure EP is mainly from the dipole orientation polarization of the polar dielectrics. With the temperature rising, the degree of molecule thermal motion rises gradually. After the curing reaction, there are fewer free-moving molecular segments in the EP matrix. The movement of the molecular segments is bound by crosslinking points. The overall movement and orientation of molecular segments are difficult to establish. At this time, EP molecular dipole orientation polarization is established via the conformational change of the molecular chain segments between the crosslinking points or restricted motion of the localized chain segments. With the temperature rising, the thermal movement ability of the EP molecular chain segments strengthens. When the temperature is high enough, the EP molecules absorb enough thermal energy, from which these molecules can overcome the potential barrier energy. The local conformation of the change or orientation of the molecular chain segments can be built. The quantity of polarized dipoles increases. At this time, when the external electric field is applied to the dielectric, the dipoles will turn along the external electric field. The dipole moment along the electric field in the dielectric unit volume increases, and the relative dielectric constant rises.

The pure EP cured epoxy resin structure is used as the basis. The structure of the other two composites is compared respectively. The MMT organic modification is the main reason to form the interface structure. After the modification, the MMT lamellar spacing is large. The compatibility differences of MMT and EP monomer molecules is small. The EP monomer molecules enter into the layers, from which the interface is formed by MMT and EP composites. Therefore, the polymerization of the EP monomer molecules between layers is the necessary condition of the organic phase generating interface. In organic MMT/EP samples, the interfacial adsorption forms between the EP matrix with layers. The movement of the molecular chain segments or lattice atoms in the organic phase and inorganic phase is mutually restricted by the interface. Under low field strength (1 × 10^6^ V/m), the electrostatic attraction in the interface is 10^3^ times higher than the electric field force from the external electric field. Even with the external electric field force, the dipoles are difficult to turn along the electric field, so the relaxation polarization weakens. The relative dielectric constant decreases, but in raw MMT/EP samples, the nanocomposites cannot form between the MMT with the EP matrix. The MMT particles disperse in the EP matrix with the shape of cluster particles. On the basis of EP molecules dipoles orientation polarization, the thermionic polarization of MMT crystal is also introduced, which increases the relaxation polarization in the dielectric. This phenomenon is particularly noticeable at high temperature. At the same time, the loss caused by relaxation polarization is also reflected in the test result.

**Figure 9:**
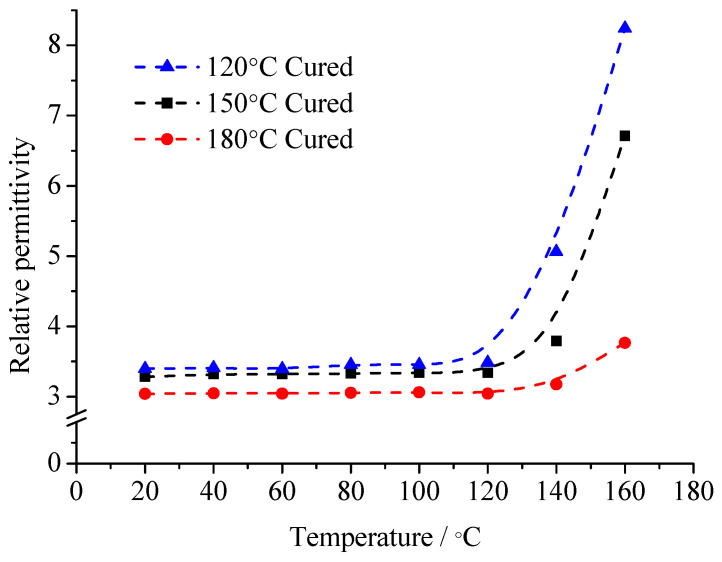
Temperature dependence of *ε*_r_ of specimens cured under various temperatures.

**Figure 10:**
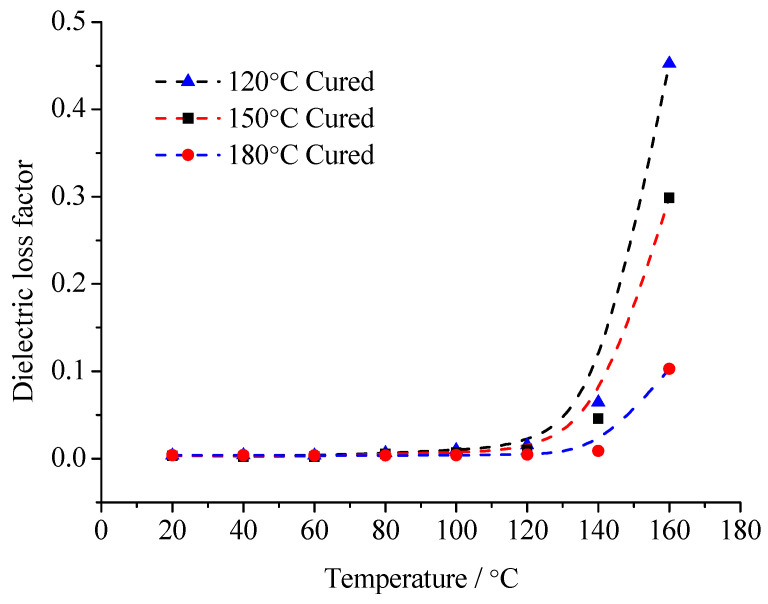
Temperature dependence of tan*δ* of specimens cured under various temperatures.

According to the consideration of the comprehensive performance index in the curing system, the pre-curing temperature of EP is 90 °C. These samples are solidified at 120 °C, 150 °C and 180 °C for 6 h. The variation curve of *ε*_r_ and tan*δ* of composites with temperature changes are shown in Figure 9 and Figure 10.

**Figure 11:**
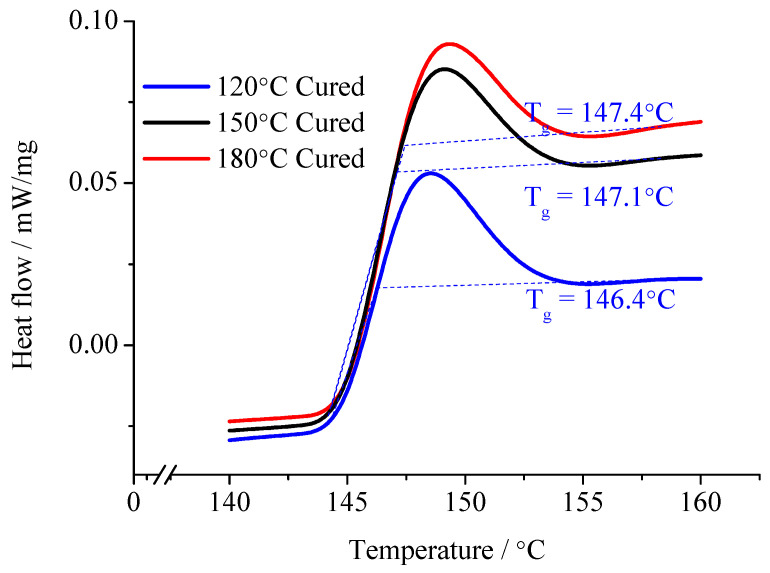
Glass transition temperature of specimens cured under various temperatures.

The composite samples were subjected to a DSC test. The glass transition temperature was obtained, from which the EP curing process at different temperatures was revealed. The test result is shown in Figure 11.

According to the relationship between composites *ε*_r_ with tan*δ*, the relaxation polarization degree, established from the sample molecules with different moving ability, can be revealed. When considering the glass transition temperature of the composites, after the curing reaction for 6 h, the difference in EP glass transition temperatures is not significant. However, the glass transition temperature of the cured sample under 120 °C is slightly lower than that of cured sample under 150 °C and under 180 °C. It illustrates that the crosslinking density of the cured sample under 120 °C is the lowest. Thus, these samples turn into microgels after precuring. Then, these samples undergo curing under different temperature. The chemical bonding can form in the microgel. Then, the crosslinking structure forms, but in cured sample under 120 °C, the crosslinking density is low. The crosslinking points are less. The molecular chain segments between crosslinking points are long. The molecular chain segments possess a certain moving ability. The stronger the moving ability of the molecular chain segments, the easier they shake off the interface adsorption. Then, the relaxation transition completes. On the contrary, when the composite interface forms between the high crosslinking density EP matrix and the MMT, the moving ability of the molecular chain segments is limited. Both the internal rotation conformation changes and local chain segments orientation are difficult to achieve. These molecular chain segments cannot shake off the interface layers restriction, so the relaxation polarization is difficult to establish.

From the test results of Figure 10 and Figure 11, in the range of 20~160 °C, the composites’ relative dielectric constant is always inversely proportional to the curing temperature. It illustrates that with low temperature curing samples, the interfacial adsorption does not easily restrict the molecular motion. The dipoles orientation polarization can be built to some extent, so the *ε*_r_ value is high. With the curing temperature rising, the crosslinking density in the EP matrix increases gradually. The molecular chain segments between the crosslinking points shorten. The moving ability of the molecular chain segments weakens. Because of the interface layer adsorption, the steering motion of the molecular chain segments is restricted. The *ε*_r_ value is low. Especially at high temperature, when the temperature is higher than the samples’ glass transition temperature, the moving ability of molecular chain segments in the low temperature curing sample improves significantly. The dipoles orientation polarization is easy to establish. Meanwhile, at high temperature, after the polymer’s molecular chain segments shake off the interface layer restriction, the restriction effect of the interface layer on the thermionic polarization in the MMT layers also disappears. The *ε*_r_ value increases obviously. On the contrary, because the crosslinking density of the samples’ molecules under high temperature curing is high, the moving ability of the molecular chain segments is limited. They cannot shake off the interface layer absorption. The polymer’s molecular chain segments’ steering is restricted by the interface layer. Meanwhile, the thermionic polarization in the MMT layers is restricted by the interface, so the *ε*_r_ value is low. Furthermore, the moving ability of the molecular chain segments is restricted by the sufficient structure of the crosslinking. At high temperature, the molecular chain segments and the MMT layer are still stuck to each other according to interface layer. The *ε*_r_ value changes little.

For the same reason, in range of 20~60 °C, the tan*δ* value of the three samples changes little when the temperature rises. The relaxation loss in different dielectrics have no significant difference. With the temperature continuously rising, the molecular chain segments in the low temperature curing samples possess a certain moving ability. The relaxation polarization establishment is not easily restricted by interface layer absorption. The relaxation loss increases. When the temperature reaches 100 °C, the loss of the low temperature curing samples has increased significantly. If the temperature increases further, the loss is greater. More and more molecular chain segments can shake off the interface restriction. In the temperature change process, the relaxation loss of the high temperature curing samples always maintains a low degree. Considering the conduction loss caused by exogenous impurity ions, when the temperature reaches 160 °C, the loss increment of the high temperature curing samples is much less than that of the low temperature curing samples.

## 5. Macro-Performance Characterisation of Nano-MMT/EP

### 5.1. Effect of MMT Contents on Composite Dielectric Relaxation

With the inclusion of nano-MMT particles, there was a gradual increase in the composite interface structure of nano-MMT/EP. This increase, to some extent, imposes restrictions on the relaxation of the two-phase composites. The resin molecules experience more constraints as the ratio of interface structures rises, leading to a decrease in polarisation intensity. Simultaneously, the MMT content also increases.

Throughout the dispersion process, the collision probability of nano-MMT nanoparticles rises, resulting in agglomeration and the formation of microparticles. Consequently, the ratio of nanocomposite interface structures decreases. It is noteworthy that the surface energy of micro-MMT microparticles was lower than that of nano-MMT nanoparticles. This variance contributes to a reduction and eventual disappearance of the two-phase binding effect between the micro-composite interfaces.

Moreover, the test temperature remained constant during the study. The determination of the relaxation polarization process was achieved by measuring the dielectric frequency spectra of the composites. The Debye equation, modified to account for the relaxation time distribution of the polymer while neglecting conductivity, was employed. The dielectric constants of the composites were calculated using Equation (2):

(2) $$\varepsilon^{*}=\varepsilon_{\infty }+\frac{\varepsilon_{s}-\varepsilon_{\infty }}{1+{\left(i\omega \tau_{0}\right)}^{1-\alpha }}$$

In Equation (2), *α* represents the dispersion of the relaxation time. A smaller *α* corresponds to a narrower relaxation time dispersion. When *α* is equal to 0, the medium demonstrates a singular relaxation time. This equation is transformed into a trigonometric function expression, allowing the calculation of the real part *ε*′ and imaginary part *ε*″ of the dielectric constant using Equations (3) and (4).

(3) $$\varepsilon^{\prime }=\varepsilon_{\infty }+(\varepsilon_{s}-\varepsilon_{\infty })\frac{1+{\left({\omega \tau }_{0}\right)}^{1-\alpha }\sin\frac{\pi \alpha }{2}}{1+2{\left({\omega \tau }_{0}\right)}^{1-\alpha }\sin\frac{\pi \alpha }{2}\;+{\left({\omega \tau }_{0}\right)}^{2(1-\alpha )}}$$

(4) $$\varepsilon^{\prime \prime }=(\varepsilon_{s}-\varepsilon_{\infty })\frac{{\left({\omega \tau }_{0}\right)}^{1-\alpha }\cos\frac{\pi \alpha }{2}}{1+2{\left({\omega \tau }_{0}\right)}^{1-\alpha }\sin\frac{\pi \alpha }{2}\;+{\left({\omega \tau }_{0}\right)}^{2(1-\alpha )}}$$

From Equations (3) and (4), *ε*′ and *ε*″ can be calculated as the reactive and active components, respectively.

(5) $$\tan\left(\delta \right)=\frac{\varepsilon^{\prime \prime }}{\varepsilon^{\prime }}$$

In Equations (3) and (4), *wt*_0_ can be eliminated, as shown in Equation (6):

(6) $${\left[\varepsilon^{\prime }-\frac{\varepsilon_{s}+\varepsilon_{\infty }}{2}\right]}^{2}+{\left[\varepsilon^{”}+\frac{\varepsilon_{s}-\varepsilon_{\infty }}{2}\tan\frac{\pi \alpha }{2}\right]}^{2}={\left[\frac{\varepsilon_{s}-\varepsilon_{\infty }}{2}\frac{1}{\cos\frac{\pi \alpha }{2}}\right]}^{2}$$

**Figure 12:**
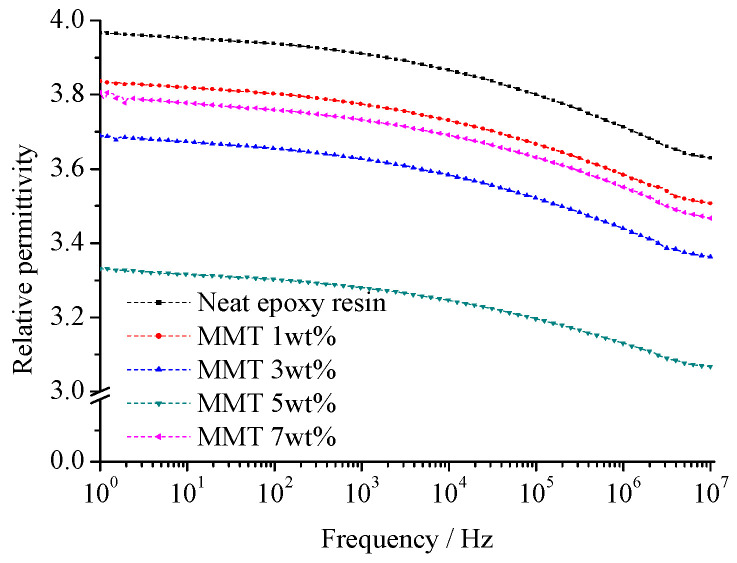
Frequency dependence of *ε*_r_ for specimens with various montmorillonite content.

**Figure 13:**
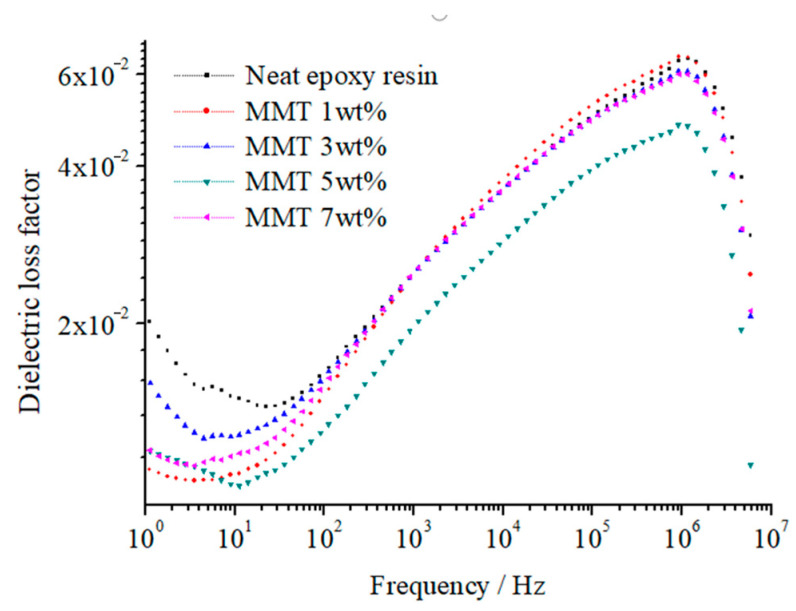
Frequency dependence of tan(*δ*) for specimens with varying montmorillonite content.

On this basis, we measured the relative dielectric constant and dielectric loss at various frequencies. The calculation of the sample relaxation time distribution parameter, denoted as *α*, at the test temperature (20 °C), was determined by solving specific equations. Figure 12 and Figure 13 illustrate the relative dielectric constant (*ε*_r_) and loss factor (tan (*δ*)) of pure EP and different nano-MMT/EPs within the 1–10^7^ Hz frequency range. The corresponding relaxation time distribution parameters for all samples are summarized in Table 3.

From Figure 12, the size of the nanoparticles is small. These particles possess great specific surface area and surface energy, which is adsorbed into the macromolecule in the polymer matrix easily. The nanoparticles interact with the macromolecule, from which the system equilibrium is achieved. When the nanoparticles mass fraction is low, the collision probability of nanoparticles with the polymer matrix is high. Because of the samples’ composite interface interaction, the polymer’s macromolecule is limited obviously. The interface structure restricts relaxation polarization establishment of two phases in the composites. With the nanoparticles mass fraction increasing, the ratio of interface structure increases. In addition to nanoparticles agglomeration, the nanoparticles disperse uniformly. The effective composite of the interface structure decreases. The effect of two-phase binding between the nanocomposite interface weakens and even disappears.

The test results depicted in Figure 12 and Figure 13 reveal that, at a constant temperature, the relative dielectric constant (*ε*_r_) of all the samples gradually decreased as the frequency increased. Based on these findings, it can be inferred that the polarization establishment process of the composites mirrored that of the pure EP, indicating a relaxation process. The range of responses for peak tan(*δ*) values across all samples remained consistent with the frequency, indicating the absence of any new polarization.

From Figure 13, in the range of 1~10^7^ Hz frequency, the composites are similar with pure EP. Only one loss peak exists. Although the values of tan*δ* are different, the corresponding response frequency of loss peak is basically identical. According to the test results, the Maxwell–Wagner interfacial polarization does not appear in the range of 1~10^7^ Hz frequency. The essence of interfacial polarization is the heterogeneous dielectric constituted by several components with different relative dielectric constant or conductivity. Under an external electric field, this heterogeneous dielectric is caused by electrons or ions accumulation in the interface. For nanocomposites, under the test voltage, the dielectric conductance is mainly ionic conductance. The dielectric is the material which has difficulty conducting electricity. In cable insulation material, the bond between atoms and molecules is stronger than that of the metallic conductor, from which the electric charge is hard to transfer, but under the effect of an applied electric field, the dielectric molecules arrange directionally. The charges move and gather between molecules, which forms the current. The dielectric intrinsic ionic conductance or electronic conductance is unlikely to happen. The source of the ions is mainly exogenous impurity ions and weakly bound ions in MMT. Under the test voltage, the average carrier mobility is low. Compared with electrons, the ions are not easily trapped, which restricts the accumulation of partial electric charges. Under alternative electric fields with a range of 1~10^7^ Hz frequency, the ionic carrier finds it difficult to accumulate in the composite interface. Therefore, in test frequencies, the dielectric loss is also mainly relaxation polarization loss. Under the effect of an applied electric field, the polar molecules or ions in the dielectric produce the phase difference owing to thermal motion. In this process, the electric energy is converted into thermal energy. It is relaxation polarization loss. The polarization process under the alternative electric fields takes a certain time, which is the relaxation time. When the frequency of the alternative electric fields and particles relaxation time matches, the phase difference will generate. Finally, relaxation polarization loss forms. There will be no new polarization in the dielectric.

As shown in Table 3, the addition of nano-MMT particles led to a decline in the relaxation time distribution parameter (*α*). Upon reaching a 5 wt.% MMT content, *α* reached its minimum value. A similar trend was observed in the changes of *ε*_r_ and tan(*δ*). It can be inferred that the effective composite interface ratio of 5 wt.% nano-MMT/ER was higher than that of the other samples. The nano-MMT particles were effectively dispersed in the ER matrix and adsorbed heterogeneously.

Beyond 5 wt.%, as the particle content increased, the collision probability of the nano-MMT layers rose, resulting in the formation of agglomerated particles and a subsequent decrease in the interface ratio.

**Figure 14:**
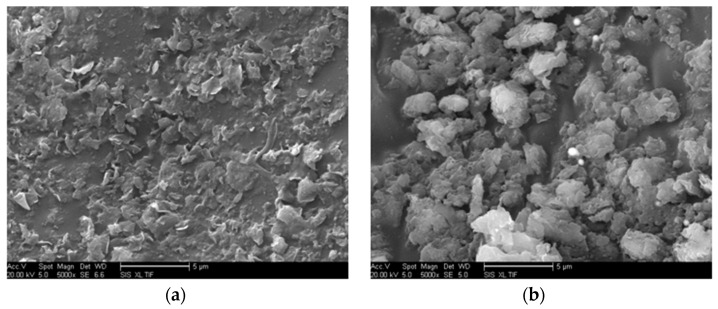
SEM image illustrating the fracture surface in specimens with (**a**) 5 wt.%, and (**b**) 7 wt.% MMT concentrations.

At 7 wt.% nano-MMT content, the *ε*_r_ and tan(*δ*) of nano-MMT/EP remained lower than those of pure EP, indicating the persistence of a partially effective nanocomposite interface in the samples with higher MMT content. The SEM images of the nano-MMT/EP samples with 5 wt.% and 7 wt.% MMT (Figure 14) revealed the agglomeration of MMT particles. Specifically, at 5 wt.%, nanoparticles were dispersed in a layered form, while at 7 wt.%, some nanoparticles were observed in clusters.

Furthermore, Figure 14 illustrates that there was only one loss peak in all the samples within a frequency range of 1–10^7^ Hz. While the tan(*δ*) values differed among the samples, the response frequencies corresponding to the loss peaks remained consistent. Test results indicated the absence of Maxwell–Wagner interfacial polarizations in the frequency range of 1–10^7^ Hz. Interfacial polarization typically arises in heterogeneous mediums composed of components with different dielectric constants or conductivities. Under an external electric field, electrons or ions of the dielectric accumulate at the interface, resulting in interfacial polarization.

In the case of nanocomposites, ionic conductivity predominates at room temperature, with minimal occurrence of dielectric intrinsic ionic and electronic conductivities. The ion sources primarily consisted of exogenous impurity ions and weakly bound MMT ions. The average carrier mobility under the test electric field was very low, making ion capture by traps challenging compared to electrons. Consequently, partial electric charge accumulation did not occur. In this manuscript, the ionic conductivity is characterized by direct measurement. Two electrodes are dipped into solution. Under the effect of an applied electric field, the current through electrodes is measured, from which the ionic conductivity can be calculated. The specific process is as follows.

(1) The conductivity meter and tested samples are prepared. The conductivity meter is in normal working condition. Furthermore, the surface of the composite samples is clean with no impurities.
(2) The electrodes in the conductivity meter are contacted with tested composites samples. They are close together, which avoids loose contact or a short circuit.
(3) The resistance value showing in the conductivity meter is read. According to the relationship between resistance and resistivity, the conductivity can be calculated. The relationship between resistance and conductivity is shown in Equation (7):

(7) σ=1/ρ, ρ=R×S/L

Among them, σ is conductivity, ρ is resistivity, R is resistance, R is the sectional area, and L is length.

For nanocomposites samples, the copper electrode is contacted with the surface of the samples, from which more accurate test results can be obtained.

Under alternating electric fields with a frequency range of 1–10^7^ Hz, carriers exhibited minimal shift towards the composite interface, and interface accumulation did not occur. Therefore, within the tested frequency range, the predominant cause of dielectric loss was relaxation polarization, with no observation of new polarization types.

### 5.2. Effect of MMT Contents on Composites Conductivity

The dielectric conductivity, *σ*, is directly proportional to carrier concentration, *n*, and carrier mobility, *μ*, as shown in Equation (8):

(8) σ=nqμ

The carrier concentration in the nano-MMT/EP is influenced by the MMT content. The introduction of MMT into the EP matrix is attributed to the dislocation lattice present in MMT. The lattice defects in MMT are comparable to those of weakly bound impurity ions, leading to charge transfer when subjected to an applied electric field (3.3 kV/mm) and resulting in the generation of a conductive current. When MMT particles are added into the EP matrix, there are two methods. One way is impure, weakly bound ions locate in interstitial site between the MMT lattice atoms. Usually, this is called interstitial impurity. The other way is that impure, weakly bound ions locate in lattice points. They replace the lattice atoms. Usually, this is called substitutional impurity. Thus, the MMT lattice defect results from impure, weakly bound ions. Ion migration in the ER matrix is induced by thermal ionic vibrations. The carrier mobility is determined by the bound potential barrier of neighboring molecules on the ions.

**Figure 15:**
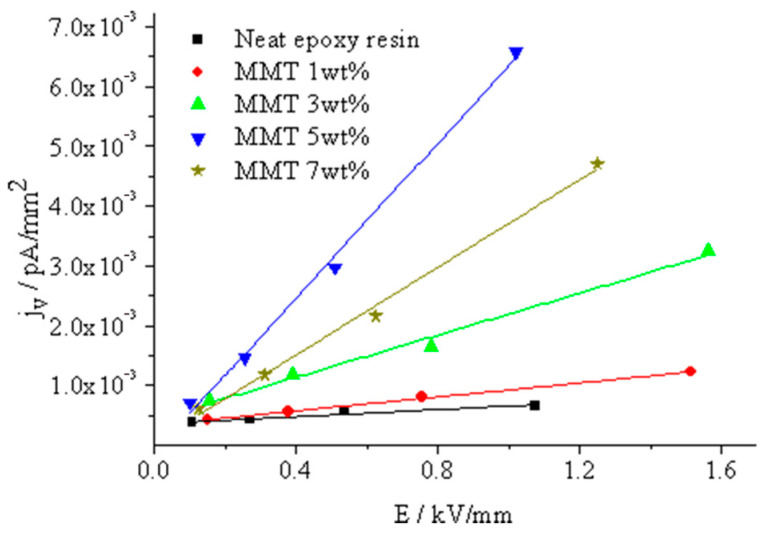
Curves depicting volume current density as a function of electric field intensity in specimens with varying MMT concentrations.

In Figure 15, the relationship between the current density and field strength for various nano-MMT/EP samples is depicted. Due to challenges in precisely controlling the sample thickness, the thicknesses are not exactly uniform. Consequently, under the same testing voltage, the electric field strengths of the samples deviate. Utilizing Equation (8), the composite conductivity can be computed using the slope illustrated in Equation (9).

(9) j=σE

The test results indicated a gradual increase in sample volume conductivity with higher MMT content. The composite conductivity peaked at almost 5 wt.% MMT content, but when increased to 7 wt.%, it declined. This phenomenon can be explained as follows.

Ionic carrier migration in the EP matrix results from ionic thermal vibration. Ions vibrate at the equilibrium position with the lowest potential energy, and adjacent resin molecules create a potential barrier to ion migration. When the ion’s thermal vibrational energy surpasses the bound potential barrier of the resin molecules, ions move from their equilibrium position, leading to charge transfer.

The addition of MMT has two effects on ion migration. Firstly, it introduces weakly bound ions into the resin, increasing ion carrier concentration with higher MMT content. Secondly, the MMT and resin matrix create a nanocomposite interface, enhancing the arrangement of the resin molecules in the interface area. In the composite interface microdomain, the bound potential barrier of the resin molecules for ion migration remains consistent, elevating the probability of ion migration.

In a pure EP matrix, polycondensation occurs between the resin monomer molecule and the curing agent CNTs-MA, from which the crosslinking structure with crosslinking points connection forms. This crosslinking structure restricts the resin molecules ordered arrangement in polymers. If the crosslinking degree is high, the crosslinking density is high. The quantity of linkages in the structural unit between crosslinking points is less. The resin molecules cannot complete the ordered arrangement according to the conformational change or molecular segments shift, from which the crystalline areas are hard to form. Even at a low crosslinking degree and crosslinking density, the quantity of linkages in the structural unit between crosslinking points is sufficient. The chain segments can complete the ordered arrangement in the local microdomain according to the conformational change or chain segment orientation. An aggregate structure similar to a polymer crystal zone forms. Compared to uncrosslinked resin or linetype polymer molecules, these aggregate structures are incomplete in space distribution order. A large number of structural defects exist. From the macro view, the space distribution of aggregation crosslinking points shows randomness. The molecules are evenly distributed in the materials, but when the observation scale extends to the ionic radius level, the molecular chain segments between these crosslinking points distribute unevenly. The intermolecular distance is different, from which every ions’ migration distance in the polymer matrix molecular array is different. The potential energy barrier that needs to be overcome is also different. The ratio of ions possessing the same thermal vibrational energy completing the migration within the matrix array is also different. The ions’ migration in crystals can be described using a double-well model. In crystals, the lattice structure possesses high regularity. The unit cell center distance is basically identical. If one ion appears within the lattice spacing, the potential energy of the ions experienced from neighbor molecules is basically the same. According to the self-thermal vibration of the ions, the barrier needing to be overcome for each ion’s migration within the crystalline lattice is also the same.

Combining the test results of Figure 13 and Figure 15, the composite interface formation can improve the distribution ordering of resin molecules in the interface area, which homogenizes the polymer intermolecular distance. The bound potential energy of polymer molecules on ions tends to be consistent, so the barrier needing to be overcome of the migration of ions in the interface microdomain is almost identical. When the ions’ thermal vibrational energy reaches a certain level, under an external electric field, the ratio of directional migration of ions in the interface increases. The trap effect is overcome. In unit time, the quantity of ions through the cross section increases. The macro average drift rate of ions in the electric field direction increases. It means the carrier mobility in the unit field strength increases. From the macro view, the dielectric conductivity increases.

### 5.3. Effect of Organic Treatment on Composite Breakdown Strength

**Figure 16:**
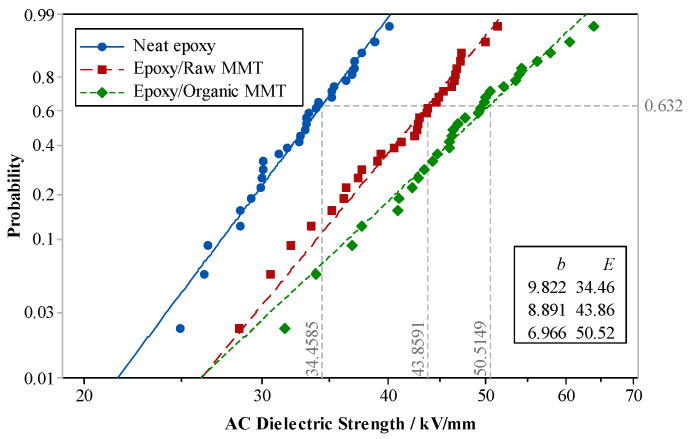
Weibull’s distribution of breakdown strength in different compound types.

The pure EP, raw MMT/EP, and nano-MMT/EP samples were subjected to a breakdown test. The two-parameter Weibull distribution of different sample breakdown strengths is shown in Figure 16.

In Figure 16, it is evident that irrespective of whether the MMT underwent organic treatment, the breakdown field strength of the composites increased to varying degrees after the incorporation of EP. Notably, the breakdown strength of nano-MMT/EP reached the highest level.

When free charges migrate within the composite dielectric, they collide with the MMT particles. Isomorphous replacement between Al^3+^ and low-valent ions leads to negative electrification of MMT lamellae. Along the electronic route, an electrostatic force opposing the electric field force in the MMT lamella arises. This force acts on free electrons, with greater strength when the distance is smaller. This electrostatic force, akin to electronic braking, diminishes energy, thereby reducing the likelihood of ionization due to high-energy electron impact. Higher field strength is necessary for ionization, compelling electrons to accumulate more energy. Consequently, the breakdown field strength of the composites surpasses that of the pure EP.

**Figure 17:**
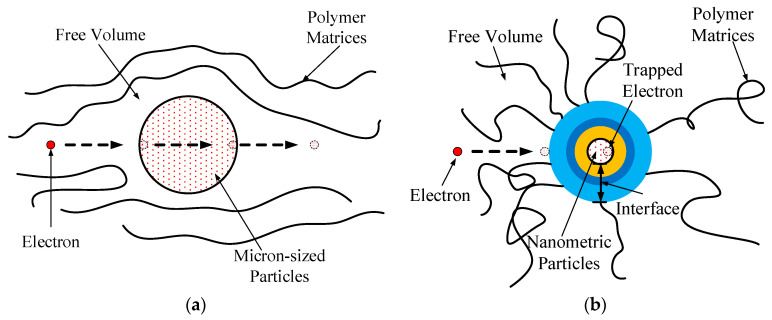
Electron migration in compound dielectrics of varying sizes. (**a**) Electron migration in micron-sized compound dielectric. (**b**) Electron migration in nanometric dielectric.

The interfacial structure of the micro-composite significantly differed from that of the nanocomposite. The nanocomposite’s interfacial structure exhibited unique physicochemical properties, resulting in a higher breakdown field strength compared to the micro-composite. This difference can be attributed to the larger spatial dimensions of the dispersed phase in the nanocomposite, creating a larger free volume in the polymer matrix. This increased free volume facilitates the accumulation of energy during electron migration. Figure 17 illustrates the electron transport processes in both the micro-composite and nanocomposite.

### 5.4. Effect of MMT Content on Composite Breakdown Strength

**Figure 18:**
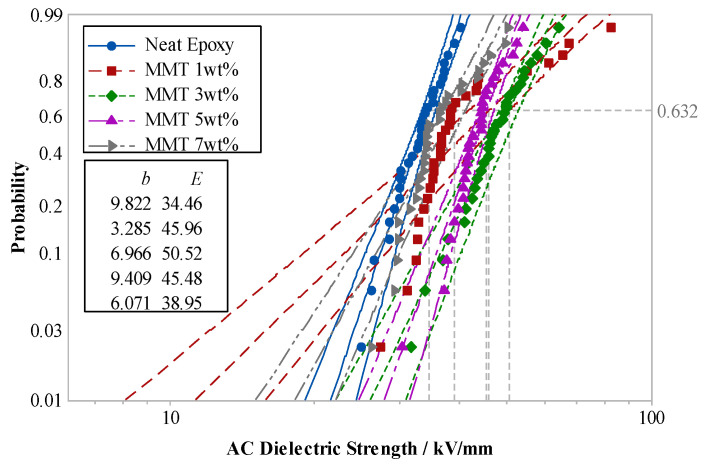
Weibull’s distribution of breakdown strength in different MMT content.

Various nano-MMT/EP samples, each with MMT contents of 1 wt.%, 3 wt.%, 5 wt.%, and 7 wt.%, along with pure EP samples, underwent power-frequency AC breakdown tests. Figure 18 illustrates Weibull’s distributions of the breakdown strengths for the two parameters.

The test results indicated that the breakdown field strength of nano-MMT/EP surpassed that of pure EP. With an increase in MMT content, the breakdown-field strength of the composite gradually rose. The maximum breakdown field strength was observed at 3 wt.% MMT content. However, as the MMT content continued to increase, the breakdown field strength started to decline. Specifically, the breakdown field strength of nano-MMT/EP with 7 wt.% MMT was 22% lower than that of nano-MMT/EP with 3 wt.% MMT.

**Figure 19:**
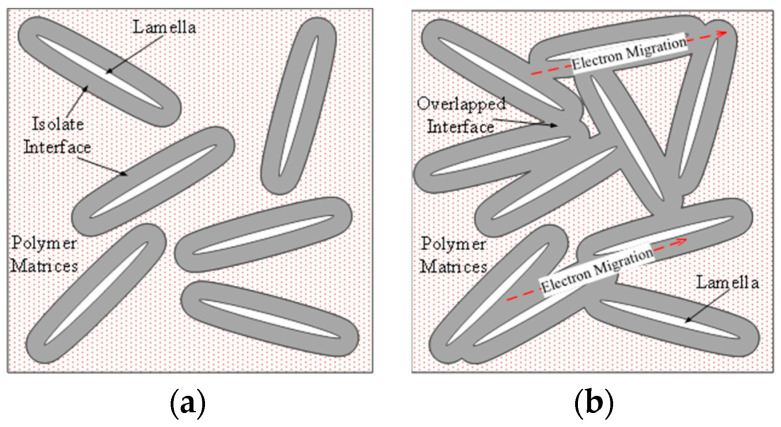
Illustration of the overlapped interface phenomenon. (**a**) Isolated interface in low MMT content composite. (**b**) Overlapped interface in higher MMT content.

This outcome can be attributed to interface overlapping, as illustrated in Figure 19. The dielectric relaxation test on nanocomposites revealed that when the MMT content reached a certain threshold, nanoparticles reaggregated in the polymer matrix, leading to interface overlapping before reaching that specific threshold.

A nanocomposite interface was established between the nano-dispersed phase and the polymer matrix, with the thickness of the nanocomposite interface typically reaching a scale of dozens of nanometers. The thickness of this interface is constrained and depends on the level of interaction between the matrix and the dispersed phase. Short-range forces resulting from strong chemical bonds, such as ionic and covalent bonds, lead to a thinner interface, while long-range forces from hydrogen bonds, van der Waals forces, and electrostatic forces result in a thicker interface.

In the breakdown field strength test, an interface overlap was observed when the MMT content was 3 wt.%. At this point, the nanometer-sized dispersive phases did not rejoin, but the interface overlap reduced the dielectric breakdown field strength. With an increase in MMT content, the distance between adjacent peeling sheets decreased, leading to a higher ratio of the overlapping interface area. When the MMT content exceeded the percolation threshold, the particles reconnected, causing the agglomerated particle size to increase and gradually destroy the nanocomposite interface. Consequently, the dielectric system underwent a transformation into a micro-composite.

From the related references, the thermal oxidative aging reaction activating energy of nano-MMT/EP with 1%, 3%, 5%, and 7% mass fraction are 47.6 kJ/mol, 50.4 kJ/mol, 52.3 kJ/mol, and 50.8 kJ/mol. It conforms to the test results in this manuscript.

## 6. Conclusions

In this study, the bisphenol-A EP served as the polymer matrix, while MMT was employed as the nanometer-scale dispersive phase. Various composite samples were prepared through melt intercalation. Through the analysis and characterization of diverse sample interface morphologies, we delved into the forming process of the nanocomposite interface. The composite insulation characteristics, encompassing dielectric relaxation, dielectric loss, electric conductance, and short-term breakdown, were tested and characterized, shedding light on the charge transport mechanisms at the composite interface. Additionally, the impact of the nanocomposite interface on the dielectric properties of the composites was investigated. On the whole, the nano-MMT particles are added into the resin matrix. The composites are formed. The ionic conductivity in the polymer is affected obviously. With the nano-MMT particles added, the carrier concentration of weakly bound ions increases. In addition, the interface of the nanocomposites increases the degree of the order of the resin molecules in the spatial arrangement. The average distance between polymer molecules is homogenized, from which the average potential barrier of ions migration in the matrix decreases. The higher the ratio of the composite interface, the lower the migration potential barrier is. Based on these experimental results, the following conclusions were drawn:

1. In the tests of the sample dielectric spectrum, as the MMT content increased, the time distribution of the composite dielectric relaxation gradually decreased, indicating an augmented interfacial structure. The number of matrix molecules bound to the interface also increased. At 5 wt.% MMT content, the relaxation time distribution was the narrowest, signifying the most pronounced effect of the interface restriction. However, at 7 wt.% MMT content, the relaxation time distribution broadened, suggesting a decline in the effect of interface restriction. The SEM test results revealed that when the MMT content exceeded 5 wt.%, MMT particles were reunited, leading to the gradual disappearance of the nanocomposite interface.
2. In nanocomposite systems, the polymer matrix’s free volume is limited due to the tight adsorption at the interface, making it challenging for electrons to attain high energies. The localized energy state in the interface layer exhibits a multilayer structure. As electrons migrate between adjacent energy states, they must surmount potential barriers or generate tunnel emissions, substantially reducing the probability of electron migration. Even through the electrons can be launched into nanoparticles, due to the small size of nanoparticles, a long and continuous conduction band would not form. When the electrons migrate in nanoparticles, they are unable to obtain acceleration to enhance their energy. They cannot be launched into the matrix. They are encumbered electronics, from which the space charges accumulate. The electronic mean free path greatly reduces, which increases the short time electrical breakdown strength. With an increase in MMT content, the distance between the dispersed phase layers decreases, causing the interfaces to overlap. In these overlapping interface areas, polymers exhibit an ordered arrangement, forming long, consecutive crystalline areas. Consequently, the electron transport path extends, facilitating the acceleration of electrons.

## Figures and Tables

**Table 1:** Experimental reagents.

Regents	Purity	Manufacturer
Natural Na-MMT	99.8%	Aladdin Reagent Co., Ltd. (Shanghai, China)
STAC	Analytical pure	GHTECH Technology Co., Ltd. (Guangzhou, China)
Silver nitrate solution	Analytical pure	Aladdin Reagent Co., Ltd. (Shanghai, China)
Bisphenol A type epoxy resin E-44	Analytical pure	Damao Chemical Reagent Factory (Tianjin, China)
3-MeH-HPA	Analytical pure	Damao Chemical Reagent Factory (Tianjin, China)
Acid anhydride	Analytical pure	Damao Chemical Reagent Factory (Tianjin, China)

**Table 2:** Experimental instruments.

Instruments	Model	Manufacturer
TEM	JEM-2010	Edinburgh Instruments (Edinburgh, Britain)
SEM	FEI Sirion 200	Thermo Fisher Scientific Technology (Waltham, MA, USA)
XRD	UV-3600	Shimadzu Corporation (Kyoto, Japan)
FTIR	CZ304	Bruker Corporation (Waltham, MA, USA)
Electronic balance	YP202N	Shimadzu Corporation (Kyoto, Japan)
Electric boosting mixer	JJ-1	Oxford Instruments Technology Co., Ltd. (Shanghai, China)
Instrument thermostatic water soluble pot	DSY-2-4	Aohua Instruments Co., Ltd. (Changzhou, China)
Heat-adjusting SHT	SHSL	Xiangyi Laboratory Instrument Development Co., Ltd. (Changsha, China)
Vacuum pump	2XZ-1	Yuhua Instruments Co., Ltd. (Gongyi, China)
Vacuum oven	DZF-6020MBE	Ohaus Corporation (Parsippany, NJ, USA)
Sand core funnel	G4	Yuhua Instruments Co., Ltd. (Gongyi, China)

**Table 3:** Values of *ε_s_*, *ε*_∞_, *ε_s_*–*ε*_∞_, and *α* for specimens with various montmorillonite content.

Montmorillonite Content/wt.%	*ε_s_*	*ε*_∞_	*ε_s_*–*ε*_∞_	*α*
0	3.99	3.51	0.48	0.748
1	3.83	3.43	0.4	0.713
3	3.75	3.35	0.4	0.726
5	3.33	3.03	0.3	0.697
7	3.78	3.42	0.46	0.718

## Data Availability

Data are contained within the article.
